# Inhibitory or excitatory? Optogenetic interrogation of the functional roles of GABAergic interneurons in epileptogenesis

**DOI:** 10.1186/s12929-017-0399-8

**Published:** 2017-12-05

**Authors:** Hui Ye, Stephanie Kaszuba

**Affiliations:** Department of Biology, Loyola University Chicago, Quinlan Life Sciences Education and Research Center, 1032 W. Sheridan Rd, Chicago, IL 60660 USA

**Keywords:** Epilepsy, GABAergic interneurons, Optogenetics

## Abstract

Alteration in the excitatory/inhibitory neuronal balance is believed to be the underlying mechanism of epileptogenesis. Based on this theory, GABAergic interneurons are regarded as the primary inhibitory neurons, whose failure of action permits hyperactivity in the epileptic circuitry. As a consequence, optogenetic *excitation* of GABAergic interneurons is widely used for seizure suppression. However, recent evidence argues for the context-dependent, possibly “excitatory” roles that GABAergic cells play in epileptic circuitry. We reviewed current optogenetic approaches that target the “inhibitory” roles of GABAergic interneurons for seizure control. We also reviewed interesting evidence that supports the “excitatory” roles of GABAergic interneurons in epileptogenesis. GABAergic interneurons can provide excitatory effects to the epileptic circuits via several distinct neurological mechanisms. (1) GABAergic interneurons can excite postsynaptic neurons, due to the raised reversal potential of GABA receptors in the postsynaptic cells. (2) Continuous activity in GABAergic interneurons could lead to transient GABA depletion, which prevents their inhibitory effect on pyramidal cells. (3) GABAergic interneurons can synchronize network activity during seizure. (4) Some GABAergic interneurons inhibit other interneurons, causing disinhibition of pyramidal neurons and network hyperexcitability. The dynamic, context-dependent role that GABAergic interneurons play in seizure requires further investigation of their functions at single cell and circuitry level. New optogenetic protocols that target GABAergic inhibition should be explored for seizure suppression.

## Background

One out of every 26 people is diagnosed with epilepsy during their lifetime, making it one of the most prevalent neurological disorders. 30% of these patients continue to have seizures despite the exhaustion of current pharmacological methods. Despite significant advances made in new pharmacological treatments, traditional anti-epileptic drugs show insufficient specificity in targeting particular cell types in the epileptic neural circuitry. The hyper-excitability of many neurons during a seizure is dynamic, demanding acute, precise temporal control of neuronal activities for effective treatment.

Optogenetic techniques are particularly suited to explore mechanisms of epileptogenesis, and could be used for future clinical treatment of seizures. The introduction of light-activated opsins can be made cell type specific, and their optical activation can be restrained precisely within a neural circuit. Optical excitation in cells can be achieved on a timescale of milliseconds, similar to that of seizure-like events. Recent studies have implemented this tool to reveal the neuronal mechanisms underlying seizures. It is possible to completely suppress seizure by optogenetic control of certain populations of neurons [1–3]. However, due to the largely unsolved complexity of seizure mechanisms, many issues still need to be addressed, including the selection of targeted cell types, its temporal precision, and optimized light stimulation parameters.

This review will focus on the functional implication of GABAergic interneurons in epileptogenesis, and current optogenetic approaches in seizure suppression with these types of neurons as the primary targets.

### Excitatory/inhibitory balance in epileptogenesis

Interactions between inhibitory and excitatory elements in a neural network shape its activity [4]. The unpredictable, synchronized firing of large populations of neurons is regarded as a consequence of an alteration in the excitatory/inhibitory balance within the neural circuitry. In support of this notion, mutations in at least 25 different human epilepsy-associated genes have been described, many of which encourage excitatory shifting [5]. Previous research indicates that hyper-excitability occurs during the transition to seizure when excitatory glutamatergic activity increases, while the inhibitory GABAergic synaptic input is weakened [6–9]. In global ischemia, both morphological and functional reorganizations happen in the CA3 network in the hippocampus. The excitatory-inhibitory balance shifts toward excitation, which leads to post-ischemic epileptiform activities [6]. In a low-Mg^2+^ model, both interneurons and pyramidal neurons in the CA1 area experience a change in intracellular signal integration during seizure transition. This is featured by the start of dominant inhibitory synaptic activity, followed by dominant excitatory synaptic activity prior to a seizure [7]. Recent studies also demonstrate alterations in various aspects of GABAergic neurons as inhibitory factors in seizure [10], which will be further discussed in the following sections.

### Traditional view of GABAergic interneurons in providing inhibitory effects to the epileptic circuitry

A frequently studied cell type in epileptogenesis is the GABAergic interneuron. By releasing the neurotransmitter gamma-aminobutyric acid **(**GABA), these neurons are traditionally regarded as inhibitory to network activity. Interactions between interneuron populations and principal cells determine the neuro-mechanism of seizure. A well-received hypothesis is that during a seizure, the ability for GABA inhibition to counterbalance membrane depolarization and action potential firing is decreased, and this modification within the interneuronal network facilitates the synchronization of the principal cells. In support of this notion, abnormalities in inhibitory GABAergic function were found in several genetic and experimental epilepsy models [11, 12]. In addition, De Lanerolle [13] reported the loss of hippocampal interneurons in human temporal lobe epilepsy (TLE). However, these anatomical changes during epilepsy alone are insufficient in determining whether GABA changes are adaptive or causal [14].

Functionally, altered GABAergic interneuron activity has been related to the synchronization and hyperexcitability of network activities in seizures [11, 15–17]. When the excitability of both parvalbumin- and somatostatin-expressing interneurons was impaired in mouse neocortex, it led to a disinhibition of the cortical network [18]. Similarly, the action potential initiation mechanism was impaired in GABAergic interneurons of a mouse model that expresses mutated human Na (V)_1.1_ gene, resulting in a hyperexcitable network [17]. When the functions of voltage-dependent sodium channels are impaired in GABAergic interneurons, it leads to reduced threshold and accelerated propagation in febrile seizures, and reduced threshold in flurothyl-induced seizures [19]. As a consequence, enhancement of GABAergic function has an anticonvulsant effect, as exhibited by the mechanism of action and efficacy of many antiepileptic drugs (AEDs).

### Optogenetic *excitation* of GABAergic interneurons for seizure suppression

In accordance with the concept that the excitatory/inhibitory balance shifts towards the excitatory regime in epilepsy, recent optogenetic studies aim to enhance the inhibitory function of GABAergic interneurons to suppress seizures. Amongst these works, Ledri et al. [3] selectively activated interneuron populations in hippocampal slices, suppressing epileptiform activity induced by 4-aminopyridine (4-AP) or by zero Mg^2+^. Interestingly, selective activation of only a subpopulation of GABAergic interneurons was not as effective in suppressing seizures. In contrast, closed-loop optogenetic activation of a subtype of GABAergic neurons, the parvalbumin (PV)-containing cells (representing 5% of hippocampal neurons) eliminated seizures in the hippocampus [1]. Ladas et al. [20] found that activating GAD-expressing interneurons with low frequency laser stimulation can attenuate epileptiform activity in the hippocampus.

A few studies combined optogenetics and stem cell transplantation technology to apply inhibitory input to the hyper-excitatory circuits. Activation of GABAergic interneuron grafts led to a suppression of pharmacoresistant seizures in the dentate gyrus (DG), due to the enhancement of synaptic inhibition in this area [21]. Cunningham et al. [22] demonstrated that human pluripotent stem cell (hPSC)-derived maturing GABAergic interneurons (mGINs) could migrate and integrate into the dysfunctional circuity of mouse brain. Using optogenetics, they found that the grafted mGINs could cause postsynaptic inhibitory responses in the host hippocampal neurons. Interestingly, these grafted neurons were already effective in suppressing seizures and ameliorating abnormalities, including cognitive deficits, aggressiveness, and hyperactivity, prior to full electrophysiological maturation.

### New view: context-dependent roles of GABAergic cells in controlling postsynaptic excitability and seizure

The traditional view that GABAergic neurons are always “inhibitory” in epilepsy is consistently challenged. The most striking evidence comes from reports suggesting that instead of being quiescent during seizure, GABAergic interneurons may be active. Interneurons (such as the somatostatin-positive subtype) can be activated in response to a 4-AP-induced seizure [23, 24]. The excitability of somatostatin-positive interneurons is higher than that of regular spiking pyramidal neurons in response to various activating stimuli, including extracellular current, low-Mg^2+^/Ca^2+^ artificial cerebrospinal fluid, metabotropic glutamate receptor agonists, and cholinergic agonists [25]. In addition, spontaneous GABAergic inhibition is increased in the soma of pyramidal neurons in temporal lobe epilepsy (TLE), although it is reduced in the dendritic regions of the pyramidal cells [26]. Thind et al. [27] further described an initial loss and later an excess growth of GABAergic synapses in dentate granule cells in a rat model of temporal lobe epilepsy. In addition, Marchionni and Maccaferri [28] showed that GABA_A_ receptor-mediated perisomatic input is enhanced during seizure. These results suggest that epilepsy might be associated with not fewer but rather abundant dysfunctional GABAergic synapses. Some authors hypothesized that these GABAergic inputs are essential in the generation of pathological, epileptic network activity [28].

At the single cell level, emerging evidence also demonstrates that functional output of GABAergic interneurons could be context dependent. GABAergic neurons can excite as well as inhibit postsynaptic neurons, depending on the states of presynaptic and postsynaptic cells. There are at least four different ways through which GABAergic interneurons could apply “excitatory” effects on network activity. (1) Raising of reversal potential. GABAergic interneurons apply excitatory input to postsynaptic principal neurons, due to an increase in the reversal potential in the principal neurons. (2) Exhaustion of presynaptic GABA. High frequency firing of the GABAergic interneuronsexhausts the presynaptic neurotransmitter GABA, which prevents the postsynaptic principal neurons from being inhibited, instead allowing for their hyper-excitability. (3) Desynchronization of the principal cells. GABAergic interneurons are responsible for the synchronized firing of principal neurons. (4) Some GABAergic interneurons inhibit other interneurons, causing disinhibition of pyramidal neurons and network hyperexcitability.

#### Raising of reversal potential (Fig. 1)

GABAergic interneurons can excite and inhibit postsynaptic neurons, depending on the GABA reversal potential in the postsynaptic cells [29, 30]. It is well known that GABA transmission depolarizes neonatal neurons owing to the high concentration of intracellular Cl^-^ at this stage [8, 23, 31–35]. The depolarizing action of GABA is not limited to neonates but can happen whenever Cl^-^ levels increase inside a segment of a mature cell [36]. For example, terminals from GABAergic axo-axonic cells contact with cortical principal neurons at their axon initial segments (AIS). They produce excitatory input to the AIS. However, there is an increased Cl^-^ gradient along the axo-somato-dendritic direction, and the reversal potential for GABA (*E*_GABA_) values decrease from the AIS to the soma and dendrites [37]. This heterogeneity of the GABA reversal potential in postsynaptic cell segments renders the spatially-distinct presynaptic inputs to generate postsynaptic responses with different magnitudes and polarities.

**Fig. 1:**
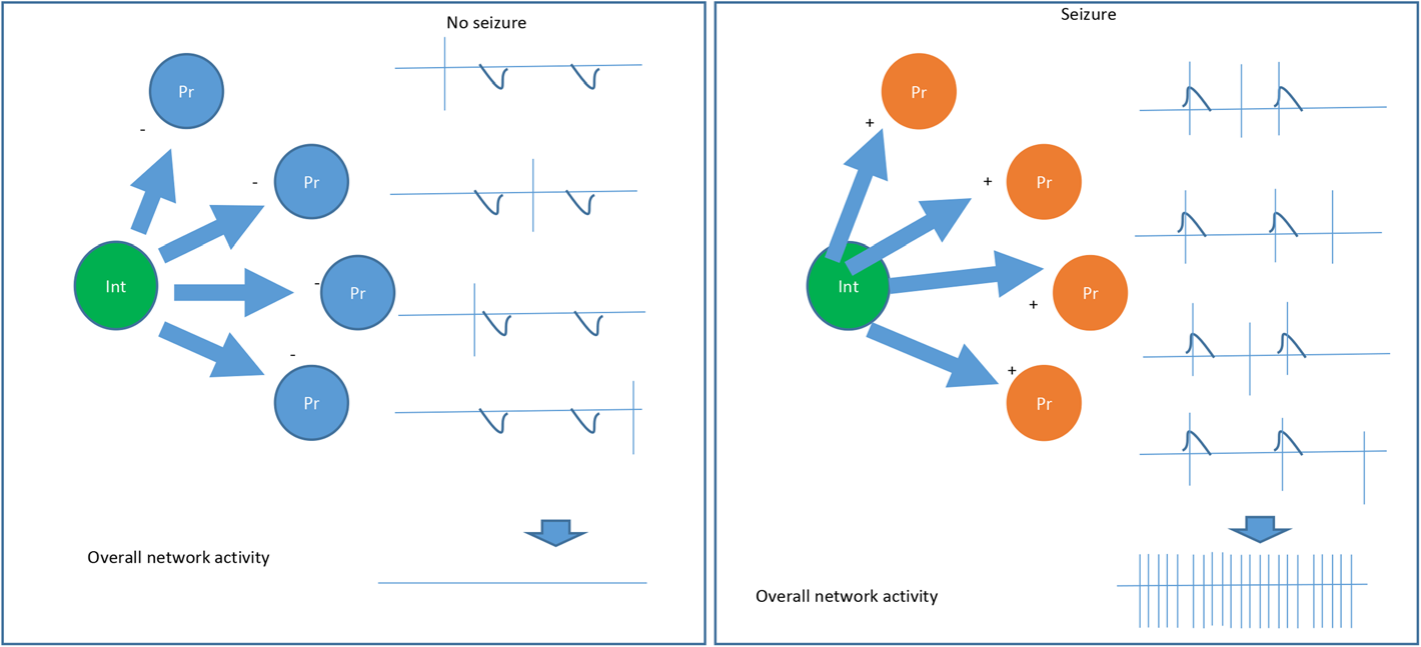
Epileptogenesis via raised reversal potential to GABAergic current. GABAergic interneurons (Int) apply excitatory synaptic input to the principal neurons (Pr) via raised GABA reversal potential in the principal neurons, which in turn increase the whole network activity and induce seizure. (+) excitatory; (−) inhibitory. Downward deflection trace: inhibitory postsynaptic potential (IPSP). Vertical line: action potential

Intracellular Cl^-^ concentration can be mediated by many cellular/molecular mechanisms. During early mammalian embryonic development, the level of Na^+^/K^+^ chloride cotransporter 1 (NKCC1) is high, and the level of potassium chloride cotransporter 2 (KCC2) expression is low [38]. This causes a high concentration of intracellular Cl^-^ and depolarization of *E*_GABA_. An increase in KCC2 expression is associated with a reduction in intracellular Cl^-^ and hyperpolarization of *E*_GABA_. Both NKCC1 and KCC2 play significant pathological roles in regulating Cl^-^ homeostasis in epileptogenesis within neonatal brain, and are proposed as potential targets for neonatal seizures [39, 40].

NKCC1 and KCC2 also play significant pathological roles in adult epileptogenesis. In drug-resistant temporal lobe epilepsy patients, up-regulation of NKCC1 mRNA was observed in the hippocampal subiculum, which contributed to the depolarized *E*_GABA_ [41]. Similarly, over-activation of NKCC1 in neurons of animal models was responsible for depolarizing *E*_GABA_, an impairing cortical inhibitory network, and triggering seizure in the presence of ammonia [42]. Following status epilepticus, upregulation of NKCC1 was observed in the deep entorhinal cortex, which contributed to the depolarizing shift of the inhibitory postsynaptic potential reversal in layer 5 neurons [43]. Genetic deletion or inhibition of NKCC1 were found to be neuroprotective against epileptogenesis [42]. NKCC1 inhibition with bumetanide prevented seizure-induced neuronal Cl^-^ accumulation and the consequent facilitation of recurrent seizures in neonatal rats [44]. Bumetanide also prevented granule cell ectopia in the dentate gyrus after febrile seizures, and the development of epilepsy [45].

In contrast to the upregulation of NKCC2 in the epileptic brain, reduction of KCC2 is another important reason for Cl^-^ accumulation in experimental [46] and human epilepsy [41, 47, 48]. In humans, KCC2 is down regulated in intractable epilepsy caused by focal cortical dysplasia [49]. Subicular pyramidal cells in patients from mesial temporal lobe epilepsy exhibit depolarizing GABA_A_R-mediated postsynaptic events, which are associated with decreased KCC2 expression [48]. In animals models, decreased KCC2 expression and impaired Cl^-^ extrusion were also found in pyramidal neurons of injured epileptogenic rat neocortex [50]. Diminished expression of KCC2 in dentate granule (DG) cells persisted for weeks in pilocarpine-induced epilepsy. This caused reduction in the inhibitory efficacy and enhancement in DG cell excitability [51]. In a mouse glioma model, the amount of parvalbumin-positive GABAergic interneurons was significantly reduced [52]. The remaining peritumoral neurons displayed elevated intracellular Cl^-^ levels and consequently, excitatory GABA responses. In these remaining neurons, KCC2 was significantly decreased. The reduced KCC2 immunoreactivity and mRNA expression [46] were associated with more positive *E*_GABA_ in epileptic tissue. The molecular mechanism for the loss of KCC2 function is related to *N*-Methyl-D-aspartic acid (NMDA) receptor activity and Ca^2+^ influx that dephosphorylate the KCC2 residue Ser940 [53].

Unbalanced NKCC1/KCC2 is not the only mechanism for intracellular Cl^-^ accumulation. When firing at high frequency, interneurons can activate the postsynaptic neurons excessively and cause chloride accumulation to depolarizing concentrations in the postsynaptic neurons, making GABA_A_ synapses excitatory [29, 54–56]. As such, GABA can provide the main post-tetanic excitatory drive to pyramidal neurons in the CA1 area of an adult hippocampus [54]. Lillis et al. [14] reported that intracellular Cl^-^ concentration largely increases in pyramidal neurons in mouse hippocampal slices during ictogenesis. Excitatory GABAergic interneurons can form a “positive feedback circuit” with the glutamatergic pyramidal cells within the strata oriens and/or pyramidale of the hippocampal CA1 region, resulting in neuronal synchronization and epileptic afterdischarge [55]. In CA3 pyramidal cells, a large depolarization in the GABA_A_ reversal potential occurs when the network enters an interictal state in a low Mg^+^/high K^+^ recurrent seizure model [57]. Clinically, the excitatory effects of GABAergic interneurons have contributed to tumor associated epilepsy [52].

#### Exhaustion of presynaptic GABA (Fig. 2)

Continuous activity in GABAergic interneurons could lead to transient GABA depletion, preventing their ability to inhibit pyramidal cells. By recording inhibitory postsynaptic currents (IPSCs) from rat CA3 pyramidal neurons in 10 mM KCl, Shin et al. [58] found that hyper-excitability in pyramidal neurons is related to the diminish of IPSCs mediated by GABA_A_ receptors. Recently, we found that high frequency firing in GABAergic interneurons could cause the exhaustion of the presynaptic neurotransmitter GABA in a low Mg^2+^/high K^+^ seizure model, therefore leading to the transition of network activity to seizure [57]. Computer simulation predicted that certain focal seizures could be triggered by GABA depletion [59]. It remains to be seen if depletion of presynaptic GABA is presented in in vivo animal models of seizure.

**Fig. 2:**
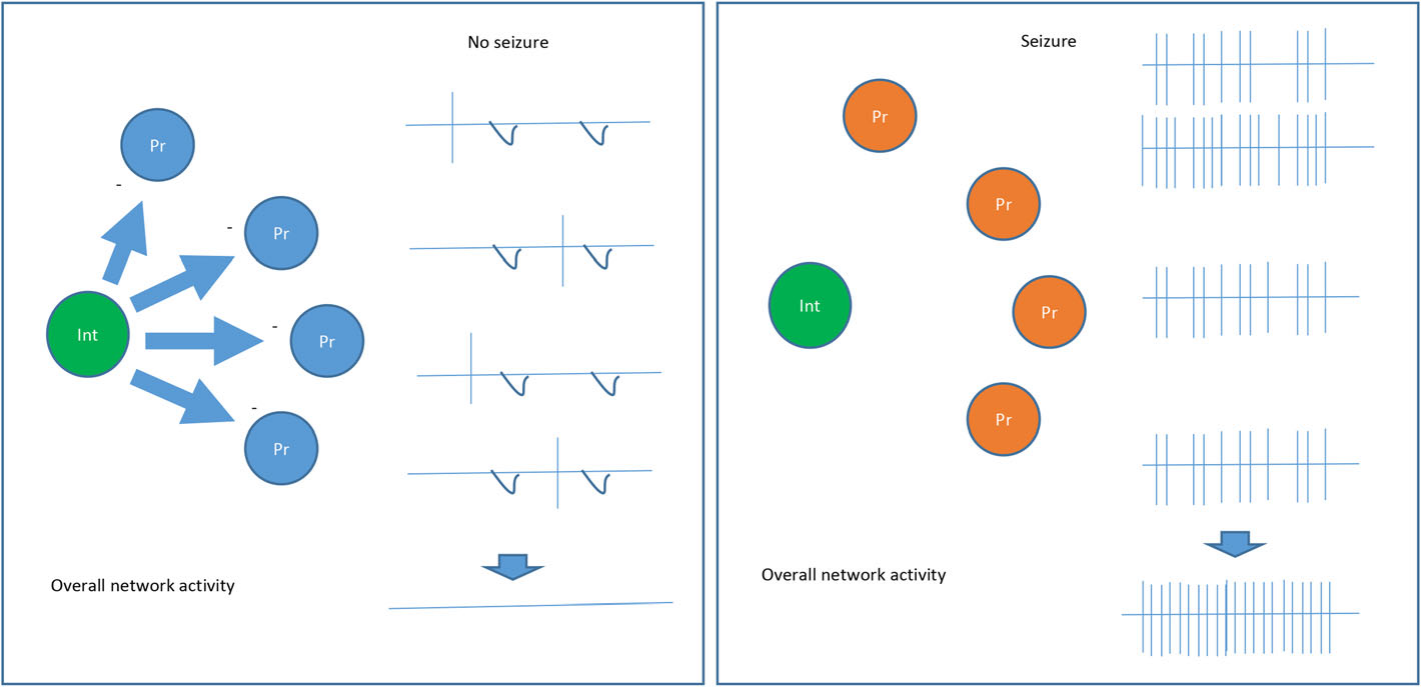
Epileptogenesis via depletion of presynaptic neurotransmitter GABA. Postsynaptic principal cell firing is enhanced due to the depletion of the presynaptic GABA and release of inhibition. (−) inhibitory

Depletion of presynaptic GABA could be monitored by the frequency of asynchronous release. Jiang et al. [60] reported that asynchronous GABA release occurs at all GABAergic synapses in fast-spiking interneurons. Asynchronous GABA release results in tonic inhibition at interneuron-principal neuron synapses in the hippocampus [61, 62]. In a genetic mouse model of epilepsy, asynchronous GABA release is found to protect the postsynaptic cell by extending the length of inhibition. Depletion of presynaptic GABA could suppress spontaneous IPSCs [63]. A substantial decrease in asynchronous GABA release results in the loss of tonic inhibition in the hippocampus of Synapsin II^−/−^ mice, prompting hyperexcitability and epileptogenesis [64]. In summary, GABA depletion decreases the inhibitory strength that interneurons apply on the principal cells.

#### Synchronization of principal cells (Fig. 3)

GABAergic interneurons can synchronize network activity during seizure [16]. First, GABAergic interneurons themselves are synchronized by gap-junctions or long-range-projections. It is likely that one individual interneuron can electrically couple to 20-50 others [65], a significant number implying that each interneuron participates in a large, continuous syncytium. Indeed, somatostatin-positive interneurons are electrically coupled via gap-junctions [25, 65, 66], which synchronize activities between coupled neurons [66] in the neocortex. Alternatively, interneurons could be synchronized by long-range-projecting GABAergic neurons from cortical areas. GABAergic neurons provide long-range, bidirectional hippocampal-entorhinal connectivity [67]. A group of long-range GABA neurons, the hippocamposeptal neurons, excite the hippocampal interneurons at the onset of epileptiform activity in immature septohippocampal formation [68].

**Fig. 3:**
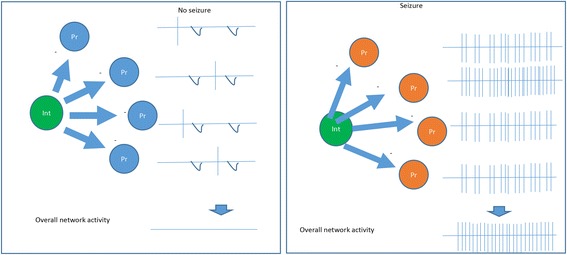
Epileptogenesis via synchronization of the principal cell (Pr) activity through GABAergic interneurons (Int). (−) inhibitory

Secondly, interneurons play a notable role in synchronizing principal cell activity and overall neural network behavior. It is found that principal cells fire synchronously with the interneurons during epileptiform discharges [69, 70]. Since each GABAergic interneuron can have contact with over 1000 pyramidal neurons in the hippocampus, these pyramidal cells may share a common temporal reference established by the same interneuron [71]. Indeed, firing of principal cells is synchronized by interneurons during high-frequency oscillation in the hippocampal network [71–74]. Furthermore, it has been shown that inhibitory interneurons synchronize the large principal neuronal population in seizure [8, 70, 75–77].

#### Disinhibition by other interneurons (Fig. 4)

GABAergic interneurons are capable of targeting other inhibitory neurons, and release these neurons’ inhibitory effects to principal cells [78, 79]. For example, when optogenetic techniques are used to activate vasoactive intestinal peptide (VIP) interneurons, it is found that VIP interneurons inhibit somatostatin and some parvalbumin interneurons, which in turn releases these neurons’ inhibition to pyramidal and principal cells [80, 81]. Owen et al. [82] demonstrated that depolarizing fast-spiking interneurons elevates the rate of GABA release, which leads to the short-term depression of inhibitory connections onto the excitatory cells in the hippocampus. In layer IV of the neocortex, fast spiking parvalbumin interneurons control pyramidal cell activity. Stimulation of somatostatin-expressing GABAergic interneurons inhibits these fast spiking interneurons, which, in turn, disinhibits pyramidal cells [79].

**Fig. 4:**
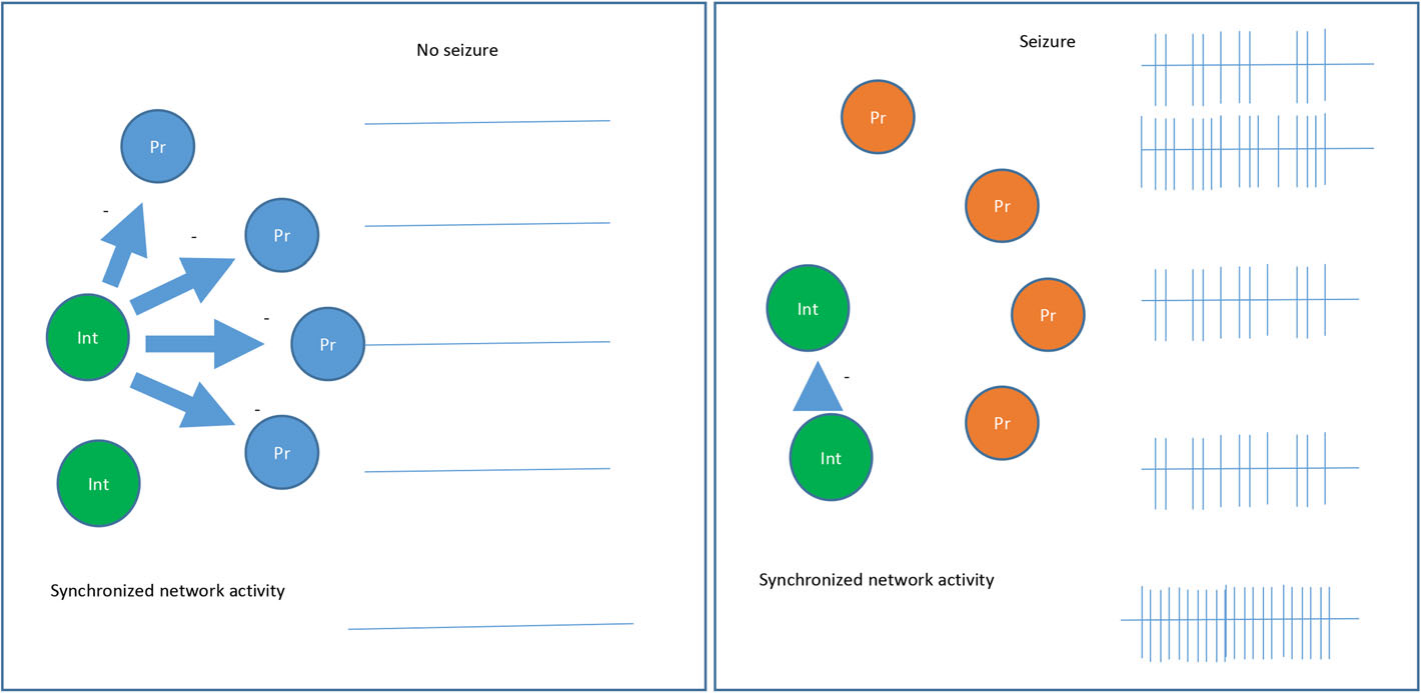
Epileptogenesis via imbibition of GABAergic interneurons. Some interneurons are inhibited by others, causing dis-inhibition of the principle cells and hyper-excitability. (−) inhibitory

The “disinhibition” hypothesis for epileptogenesis implies that removal of inhibition to the pyramidal cell could cause excitability and seizure susceptibility [83, 84]. For example, deficits in δ-subunit expression in the GABA_A_ receptor of GAD65-positive interneurons result in a reduction of the tonic inhibition to these neurons. Disinhibition of interneurons results in decreased seizure susceptibility [84]. It is unknown if one can initiate seizure by enhancing GABAergic inhibition to certain interneurons, and in turn, removing these interneurons’ inhibitory effects on the principal cells. Furthermore, it is unknown if optogenetics can be used to subdue seizure, by suppressing GABAergic interneuron activity, which permits the inhibitory effects of other interneurons be fully applied to principle cells.

### Optogenetic *inhibition* of GABAergic interneurons for seizure suppression

The context-dependent, excitatory roles that GABAergic interneurons can play, suggest a novel optogenetic strategy for seizure suppression. The widely used protocol that aims at “exciting” GABAergic neurons, is probably not optimal. Instead, this evidence begs for the investigation of seizure suppression by *inhibiting* these neurons. Using vGAT:ChR2-eYFP mice (expressing ChR2 under the interneuron-specific mouse vesicular GABA transporter (vGAT) promotor) and a local 4-AP microinjection seizure model (performed in the somatosensory cortex), Dufour and Valiante [85] found that optical activation of GABAergic interneurons could lead to seizure. The researchers speculated that the effects of the GABAergic interneurons are context dependent, contingent on the brain activity state. This observation started to challenge the traditionally accepted inhibitory effects of GABAergic interneurons in seizure. Unfortunately, the authors have not tested if optogenetic inhibition of GABAergic interneurons can suppress seizure.

**Fig. 5:**
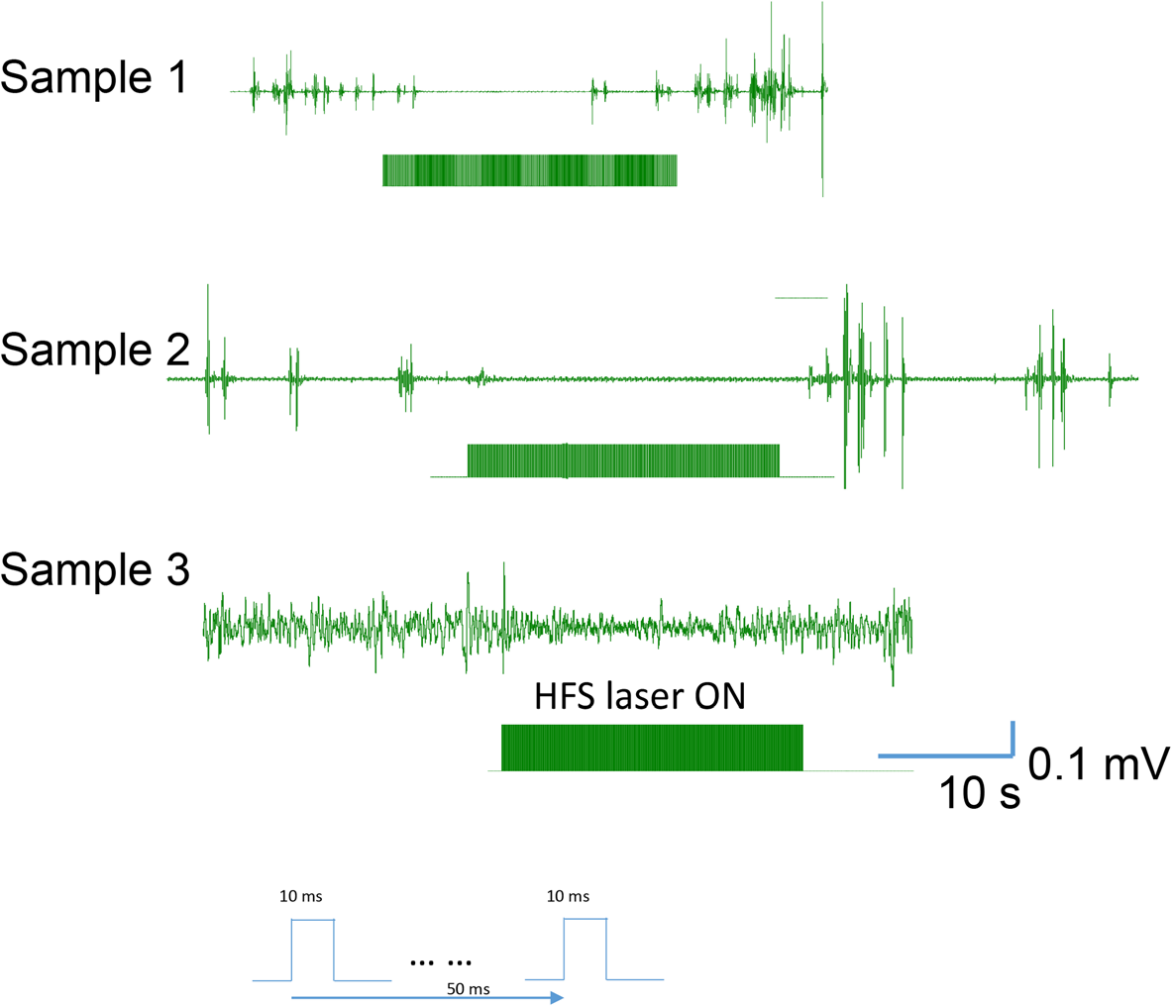
High frequency laser trains are effective in suppressing 4-AP seizure in hippocampal CA3 area in vivo. Bar: High frequency laser stimulation (20 Hz, 10 ms pulse width and 20s duration). Examples 1 and 2: complete seizure EEG suppression. Example 3: Partial suppression of seizure activity. Overall, 72.1% of the HFS trial are effective in seizure EEG suppression

We utilized a Gad2-Cre recombinase mouse line and injected an adeno-associated viral vector (AAV5-EF1α-DIO-NpHR3.0-eYFP, University of North Carolina vector core facility) into the CA3 area in the hippocampus (2 months old), resulting in expression of the light-sensitive chloride pump halorhodopsin (NpHR) in GABAergic interneurons. The functional role of GABAergic interneurons is investigated in a 4-AP seizure model (6 mg/Kg i.p. injection, five animals) by optically inhibiting these neurons. Seizure-like activity was observed 10-15 min after 4-AP injection. In early approaches, we applied continuous laser inhibition (1 min in duration, adapted from [86]) to the GABAergic interneurons. We observed subtle but quantifiable suppression of electroencephalogram (EEG), which can only be identified through a complicated EEG detection algorithm [87]. To improve the success rate of seizure suppression, we used a high-frequency stimulation (HFS) protocol [88] to inhibit GABAergic interneurons in the CA3 area during 4-AP seizure. We found that 10 ms laser pulses are effective in seizure suppression. In total, we applied 43 HFS (laser train duration 20 s, frequency 20 Hz, pulse width 10 ms, intensity 15 mW/mm^2^) in 5 different Gad 2 mice. We found that 31 (72.1%) of these HFS trains were effective or partially effective in suppressing seizure EEG. 11 (25.6%) of the trials were not effective, and 1 (2.3%) corresponded with enhanced seizure activity. Figure 5 shows several examples when seizure EEG was suppressed when GABAergic interneurons were inhibited with HFS. We are currently exploring the optimal parameters to further improve the success rate for seizure suppression, and investigate the cellular mechanism of such suppression.

## Conclusion

GABAergic neurotransmission has been traditionally regarded as inhibitory to neural network activity, and the idea that failure of GABA inhibition contributes to seizure has been dominant, and sometimes presumable. The context-dependent, possibly “excitatory” roles that the GABAergic interneurons can play in epileptic tissue, begs for the reassessment of their contribution using optogenetic tools, which can provide precise spatial and temporal control of neuronal activity with excellent resolutions. New optogenetic protocols aimed at “inhibiting” GABAergic interneurons should be explored to investigate the possibility of seizure suppression.

## Abbreviations

4-AP: 4-aminopyridine
AEDs: Antiepileptic drugs
AIS: Axon initial segments
DG: Dentate granule
*E*_GABA_: Reversal potential for GABA
GABA: Gamma-Aminobutyric acid
HFS: High-frequency stimulation
hPSC: Human pluripotent stem cell
IPSCs: Inhibitory postsynaptic currents
KCC2: Potassium chloride cotransporter 2
mGINs: Maturing GABAergic interneurons
NKCC1: Na^+^/K^+^ chloride cotransporter 1
NMDA: *N*-Methyl-D-aspartic acid
NpHR: Halorhodopsin
PV: Parvalbumin
TLE: Temporal lobe epilepsy
vGAT: Vesicular GABA transporter
VIP: Vasoactive intestinal peptide
